# Skin Cancer Narratives on Instagram: Content Analysis

**DOI:** 10.2196/34940

**Published:** 2022-06-02

**Authors:** Basma Gomaa, Rebecca F Houghton, Nicole Crocker, Eric R Walsh-Buhi

**Affiliations:** 1 Department of Applied Health Science Indiana University School of Public Health Bloomington, IN United States

**Keywords:** digital health, social media, skin cancer, Instagram, melanoma, oncology, cancer, skin, content analysis, narrative, information sharing, online platform

## Abstract

**Background:**

Skin cancer is among the deadliest forms of cancer in the United States. The American Cancer Society reported that 3 million skin cancer cases could be avoided every year if individuals are more aware of the risk factors related to sun exposure and prevention. Social media platforms may serve as potential intervention modalities that can be used to raise public awareness of several diseases and health conditions, including skin cancer. Social media platforms are efficient, cost-effective tools for health-related content that can reach a broad number of individuals who are already using these spaces in their day-to-day personal lives. Instagram was launched in 2010, and it is now used by 1 billion users, of which 90% are under the age of 35 years. Despite previous research highlighting the potential of image-based platforms in skin cancer prevention and leveraging Instagram’s popularity among the priority population to raise awareness, there is still a lack of studies describing skin cancer–related content on Instagram.

**Objective:**

This study aims to describe skin cancer–related content on Instagram, including the type of account; the characteristics of the content, such as the kind of media used; and the type of skin cancer discussed. This study also seeks to reveal content themes in terms of skin cancer risks, treatment, and prevention.

**Methods:**

Through CrowdTangle, a Facebook-owned tool, we retrieved content from publicly available accounts on Instagram for the 30 days preceding May 14, 2021. Out of 2932 posts, we randomly selected 1000 posts for review. Of the 1000 posts, 592 (59.2%) met the following inclusion criteria: (1) content was focused on *human* skin cancer, (2) written in English language only, and (3) originated from the United States. Guided by previous research and through an iterative process, 2 undergraduate students independently coded the remaining posts. The 2 coders and a moderator met several times to refine the codebook.

**Results:**

Of the 592 posts, profiles representing organizations (n=321, 54.2%) were slightly more common than individual accounts (n=256, 43.2%). The type of media included in the posts varied, with posts containing photos occurring more frequently (n=315, 53.2%) than posts containing infographics (n=233, 39.4%) or videos (n=85, 14.4%). Melanoma was the most mentioned type of skin cancer (n=252, 42.6%). Prevention methods (n=404, 68.2%) were discussed in Instagram posts more often than risk factors (n=271, 45.8%). Only 81 out of 592 (13.7%) posts provided a citation.

**Conclusions:**

This study’s findings highlight the potential role of Instagram as a platform for improving awareness of skin cancer risks and the benefits of prevention practices. We believe that social media is the most promising venue for researchers and dermatologists to dedicate their efforts and presence that can widely reach the public to educate about skin cancer and empower prevention.

## Introduction

### Background

Skin cancer, in general, and melanoma, specifically, are among the deadliest forms of cancer in the United States [1,2]. The number of individuals in the United States diagnosed with skin cancer has increased over the last 30 years [1]. Individuals aged 15-39 years have seen more deaths from melanoma in this time frame because of the growing trends in the use of tanning services and increased popularity of tanned skin [2]. The American Cancer Society reported that 3 million skin cancer cases could be avoided every year if individuals are more aware of the risk factors related to sun exposure and other forms of prevention [3].

Social media platforms may serve as potential intervention modalities that can be used to raise public awareness of several diseases and health conditions, including skin cancer. Social media platforms are efficient, cost-effective tools for health-related content that can reach a broad number of individuals who are already using these spaces in their day-to-day personal lives [4]. Additionally, social media provides a feedback loop, enabling researchers to access users’ online conversations regarding their specific needs, ultimately allowing messages developed in public health campaigns to align with those needs [5].

Instagram was launched in 2010, and it is now used by 1 billion users, of which 90% are under the age of 35 years [6]. These users generate 95 million posts each month in addition to 3.5 billion “Likes” each day [6]. In a recent study investigating the potential of various social media platforms to advance skin cancer awareness, Instagram had the greatest number of skin cancer–related posts [7]. Additionally, the hashtag #SunDamage was in the top 20 hashtags associated with dermatology-related content on Instagram [8]. Moreover, trends discussing skin cancer prevention dominate among others on Instagram, signifying its potential to specifically raise awareness about prevention among young people who may be susceptible to content promoting risky skin health behaviors such as tanning [9]. However, existing research results have limited generalizability due to the restricted study period, keywords, and sample size (eg, 150 posts) [6].

Social media is becoming a potential intervention modality to raise skin cancer awareness, especially via image-based platforms [10,11], such as Instagram. However, there is still a need to conduct additional research addressing the potential as well as the drawbacks of social media for raising public awareness regarding skin cancer [11]. This study fills this gap by describing skin cancer–related content on Instagram, including the origin (ie, source characteristics) and attributes (ie, content characteristics) of these social media posts. The study also seeks to reveal content themes in terms of skin cancer risks, treatment, and prevention.

### Research Questions

Accordingly, the following research questions (RQs) were proposed:

- RQ1: What are the source and content characteristics of Instagram posts related to skin cancer?
- RQ2: To what extent are different types of skin cancer covered on Instagram?
- RQ3: How do messages frame causes and solutions regarding skin cancer causes, treatments, and prevention?
- RQ4: To what extent do Instagram posts on skin cancer address the susceptibility and severity of skin cancer; benefits and barriers associated with diagnosis, prevention, and treatment; call to action; and readers’ self-efficacy?

## Methods

### Data Collection

We used CrowdTangle [12], a tool owned and operated by Facebook, which tracks the engagement of the publicly available content on Facebook pages, subreddits, and Instagram accounts. Through CrowdTangle, we retrieved content from publicly available accounts on Instagram for the 30 days preceding May 14, 2021. Only posts written in the English language from users based in the United States were included. Guided by previous research [9], we selected the following keywords to locate relevant posts on Instagram: “skin cancer” OR “melanoma” OR “basal cell carcinoma” OR “squamous cell carcinoma” OR “skin cancer awareness” OR #skincancer OR #melanoma OR #basalcellcarcinoma OR #squamouscellcarcinoma OR #skincancerawareness.

The initial search for our main sample yielded 2982 posts. We reordered the full set of posts descending from the highest number of “total interactions,” defined by CrowdTangle as an indicator of engagement—total reactions, comments, and shares combined. We selected the top 1000 posts in terms of total interactions, and then used Research Randomizer [13] to produce a file with numbers 1 through 1000 in a random order. By merging this file with the sample of 1000 posts, each post was assigned a study number. By randomly ordering the sample of 1000 posts, this ensured that each coder would be assigned a set of posts with a variety of total interactions.

The following inclusion criteria for post content were assessed by the coders: (1) directly related to human skin cancer, (2) in the English language only, and (3) originating from the United States. Content mentioning skin cancer in animals was excluded. Of the sample of 1000 posts, 408 (40.8%) did not meet the inclusion criteria, resulting in an analytic sample of 592 (59.2%) Instagram posts.

### Codebook Development

Although there has been increased interest in applying machine learning methods to the analysis of social media data, it has also been suggested that these techniques may present challenges when applied to qualitative coding in social science research [14]. For this study, we developed a codebook through an iterative process, guided by previous social media content analytic research. The codebook included variables related to source and post characteristics, as well as the type of media used. Coded source characteristics included whether the Instagram account or profile represented an individual or organization. Individual profiles were coded according to self-identification (eg, influencer, parent, business owner, dermatologist, and esthetician). For organizational accounts, organization type was coded using information displayed in the user’s profile or links embedded in the user profile or bio to the organization’s website (eg, business, media outlet, health care organization, and nonprofit). [9,14]. Subsequently, the content was coded into 3 categories depending on whether it addressed (1) risk factors, (2) prevention, or (3) treatment [9,15]. For the citation source, we followed the classifications used by Walsh-Buhi et al [16] from a prior content analytic study that analyzed Instagram posts. Citation sources were classified as a cancer organization, health website, celebrity, the Centers for Disease Control and Prevention, or World Health Organization. A copy of the full codebook can be found in Multimedia Appendix 1.

### Pilot

Coding was conducted independently by 2 trained undergraduate students, in multiple stages, to assess the reliability of the codebook constructs. As a precursor to coding the final sample, the coders underwent a pilot stage, in which posts from the time period of February 24 to March 25, 2021, were downloaded. A total of 1173 posts were downloaded and sorted in descending order by “total interactions.” The first 60 posts were used for coder training purposes and to provide additional insight on the reasons for exclusion/inclusion. Once this initial training took place, 35 posts were reviewed and coded by the 2 raters, of which 16 posts met our inclusion criteria. The 2 raters and a moderator met and held a discussion for 1-2 hours every 2 weeks to identify problematic variables and reach perfect agreement [17]. The codebook was revised following this pilot phase to incorporate feedback from the raters and make the definitions more accurate.

### Coding of the Main Sample

The sample for the main study was downloaded from CrowdTangle, including posts from the 30 days preceding May 14, 2021, as described above. The 2 undergraduate raters independently coded 250 posts each. Each rater double-coded 10% (n=25) of posts from the other rater’s sample of 250 posts. Of the 50 posts double-coded by the raters, 45 met the inclusion criteria. Interrater reliability was assessed using Cohen κ statistics to determine the level of consistency in codes between each rater. Of the 66 codebook variables for which a Cohen κ could be calculated, 82% (n=54) of the variables resulted in a moderate or higher agreement. The median Cohen κ was 0.78, reflecting substantial agreement between the raters on identifying codebook constructs [17]. Another meeting was held to discuss and resolve the discrepancies and address problematic variables, resulting in a final codebook. The remaining study sample posts (n=500) were then coded.

### Ethical Considerations

Institution Review Board approval was not required as CrowdTangle only imports data from public accounts on Instagram.

## Results

### RQ1: What Are the Source and Content Characteristics of Posts Related to Skin Cancer?

Of the 592 posts that met the inclusion criteria, the sources of content originated from 2 different types of profiles. Profiles representing organizations (n=321, 54.2%) were slightly more common than individual accounts (n=256, 43.2%). For posts that could not be clearly classified as being derived from either an individual or organization, an “other” category (n=15, 2.5%) was selected.

As displayed in Table 1, influencers such as public figures or celebrities were more commonly represented in this sample of posts as a source of skin cancer information than physicians, dermatologists, and other sources. Although in the case of organizational profiles, those in the “Business” category (222/321, 69.2%) led over those with medical criteria such as health organizations and health information provider. The type of media included in the posts varied. Posts containing photos (315/592, 53.2%) were more common than posts containing infographics (233/592, 39.4%) or videos (85/592, 14.4%).

For posts that could not be clearly classified as being derived from either an individual or organization, an “other” category (15/592, 2.5%) was selected, and these posts were not included in Table 1.

**Table 1:** Source characteristics of skin cancer–related posts on Instagram (N=577).

Source characteristic^a^			Post, n (%)
**Content in the bio/profile or post of an individual (n=256)**			
	Influencer or public figure	152 (59.4)	
	Physician	107 (41.8)	
	Dermatologist	83 (32.4)	
	Parent	51 (19.9)	
	Business owner	37 (14.4)	
	Journalist	14 (5.5)	
	Esthetician	11 (4.3)	
	Nurse or other health worker	4 (1.6)	
	Health educator	2 (0.8)	
**Content in the bio/profile or post of an organization (n=321)**			
	Business	222 (69.2)	
	Health information provider	53 (16.5)	
	Nonprofit	39 (12.1)	
	Health care organization	34 (10.6)	
	News organization	8 (2.5)	
	Government entity	5 (1.6)	
	School	1 (0.3)	

^a^Source characteristics for each broader type of profile (individual or organization) exceed 100% when added together because multiple categories may have been selected for a particular post (eg, a business and a health care organization).

### RQ2: To What Extent Are Different Types of Skin Cancer Covered on Instagram?

More than half (318/592, 53.7%) of the posts analyzed mentioned skin cancer generally but did not specify the type (Table 2). Melanoma (252/592, 42.6%) was the most mentioned type of skin cancer.

**Table 2:** Posts mentioning types of skin cancer on Instagram (N=592).

Type of skin cancer	Post, n (%)
Skin cancer mentioned generally, but type was not specified	318 (53.7)
Melanoma	252 (42.6)
Basal cell carcinoma	29 (4.9)
Squamous cell carcinoma	29 (4.9)

### RQ3: How Do Messages Frame Causes and Solutions Regarding Skin Cancer Causes, Treatments, and Prevention?

Just under half of all posts (271/592, 45.8%) included information regarding some kind of skin cancer risk factor. The “sun” was coded as the top named risk factor for skin cancer (227/271, 83.8%), followed by artificial tanning (eg, indoor tanning; 48/271, 17.7%) and genetics (15/271, 5.5%).

Information regarding prevention methods (404/592, 68.2%) was included in Instagram posts more often than risk factors (271/592, 45.8%). Within prevention methods, wearing sunscreen (280/404, 69.3%) was the most commonly mentioned method, followed by getting checked by a physician (131/404, 32.4%) and wearing protective gear/clothes (101/404, 25%; Table 3).

**Table 3:** Posts discussing prevention methods of skin cancer (N=404).

Prevention method	Post, n (%)
Wearing sunscreen	280 (69.3)
Getting checked by a physician	131 (32.4)
Wearing protective gear/clothes	101 (25)
Self-examination	50 (12.4)
Staying away from the sun	41 (10.1)
Mentioning warning signs	37 (9.2)
Not using tanning beds	21 (5.2)
Using self-tanning products	19 (4.7)

### RQ4: To What Extent Do Instagram Posts on Skin Cancer Address the Susceptibility and Severity of Skin Cancer; Benefits and Barriers Associated With Diagnosis, Prevention, and Treatment; Call to Action; and Readers’ Self-efficacy?

Table 4 displays the different type of posts revealed in the analyses. Posts addressing the benefits of skin cancer prevention (120/402, 29.9%) and encouraging readers to adopt a certain behavior (209/592, 35.3%) were heavily mentioned compared to posts discussing the prevalence (96/592, 16.2%) and the seriousness (75/592, 12.7%) of skin cancer. Only 4.9% (29/592) of the posts highlighted a specific diagnostic method. Of these 29 posts, 4 (14%) explained the specific benefit of the diagnostic method. The results showing the top mentioned hashtags are displayed in Table 5. Only 13.7% (81/592) of the posts provided some type of citation. Cancer organizations, health websites, and physicians were the top sources of citations, as displayed in Table 6.

**Table 4:** Types of content portrayed in Instagram posts.

Content type^a^	Post, n (%)
Information urging readers to adopt a certain behavior (N=592)	209 (35.3)
Benefits of skin cancer prevention (n=402)	120 (29.9)
Prevalence of skin cancer (N=592)	96 (16.2)
Seriousness of skin cancer (N=592)	75 (12.7)
Diagnostic method (N=592)	29 (4.9)
Benefit of diagnostic method (n=29)	4 (13.8)

^a^Due to skip logic in the extraction survey, some posts were not rated for certain content types.

**Table 5:** Top 10 hashtags appearing in the study sample posts (N=592).

Hashtag	Number of mentions, n (%)
#skincancerawareness	165 (27.9)
#skincancer	134 (22.6)
#skincare	98 (16.6)
#melanoma	89 (15)
#sunscreen	88 (14.9)
#skincancerawarenessmonth	87 (14.7)
#melanomaawareness	78 (13.2)
#melanomamonday	76 (12.8)
#spf	67 (11.3)
#dermatology	65 (11)

**Table 6:** Types of sources cited in posts (N=81).

Citation source	Post, n (%)
Cancer organization	34 (42)
Health or web source (eg, WebMD)	21 (26)
Physician	14 (17)
Research community	11 (14)
Centers for Disease Control and Prevention or federal organizations	5 (6)
Celebrity	3 (4)
World Health Organization	2 (2)

## Discussion

### Principal Findings

This study aimed to describe the content landscape of skin cancer on Instagram, specifically focusing on the content source and type of information posted by users. The study also reveals the themes of the posts in terms of skin cancer causes, diagnosis, and prevention methods. To the best of our knowledge, this is the first study to identify the sources of skin cancer content on Instagram.

Overall, slightly more content originating from organizational accounts was posted than content from accounts owned by individuals. Business-owned accounts (eg, Skin Store, Baby Bum) tended to post about skin cancer more than those of a medical background, such as health organizations or health information providers. Among individual account owners, influencers (not from a medical background) posted skin cancer–related content more than individuals who possessed medical expertise (eg, dermatologists, other physicians). The term *influencer* is considerably newer and can be defined as individuals who purposely use social media for advertising specific products and services. Influencers establish trust with their followers over time and successfully market to their customer base, leading to the presence of a new marketing method known as Influencer Marketing [18]. It is noteworthy to acknowledge the partnership between the beauty industry and social media in general [19], as celebrities and influencers play an important role in advertising for the beauty industry.

Although sunscreen promotion was the most commonly identified prevention method represented in Instagram posts (that discussed skin cancer prevention methods), such posts also contained other prevention methods included in prevention guidelines [20]. For example, getting a checkup from a physician, wearing protective clothes, and self-examinations were included in 33%, 25.4%, and 12.6% of the posts, respectively. Interestingly, other important prevention methods were infrequently mentioned in the reviewed posts, representing missed opportunities. For instance, one of the more critical prevention methods—avoiding ultraviolet (UV) radiation—was mentioned as staying away from the sun and not using tanning bed in only 10.3% and 4.8% of the posts, respectively. As exposure to UV radiation—from outdoor (ie, sun) and indoor (ie, tanning beds, lamps, or booths) light—is the most important risk factor for skin cancer [21,22], including a prevention focus in these areas might be beneficial in future skin cancer campaigns or interventions on social media. Moreover, as indoor tanning is prevalent among younger adults and women [23], it is critical that social media campaigns or interventions also focus their prevention messaging on tanning bed use.

Given that the majority of the posts originated from nonmedical accounts, it is concerning that only 13% of the posts in this sample cited their information from a credible source, such as a cancer organization. A behavioral intent study found that, of its participants, 91% said that online communities (such as Instagram) play a role in their health decisions [24]. Considering the percentage of people who report using these online platforms for health decisions, the scarcity of credible information seen in this sample is concerning. Misinformation on social media continues to be a public health challenge that needs to be addressed and combated [25] in future research.

### Comparison to Prior Work

In contrast to skin cancer content on Pinterest [14] and in Facebook groups [26], a higher percentage of posts in our sample included content about prevention and encouraging others to wear sunscreen than those discussing the risk factors. This aligns with previous research on Instagram and YouTube [9,27]. For example, Basch and Hillyer [9] similarly found a higher proportion of messages focused on prevention than risk, and they speculated that Instagram could serve as a health promotion tool, particularly among adolescents and young adults. This highlights the importance of studying individuals’ behaviors on Instagram, and it is worth extending similar inquiries to other social media platforms as well.

### Limitations

As with other research, this study is not devoid of limitations. First, we were only able to collect information from publicly available Instagram accounts. Second, our inclusion criteria may have limited the generalizability of our results. For example, we only included content in English. We also excluded profiles that mentioned they were originating from a non–US-based location. A final potential drawback of this work may be limiting the study to only a single time period within the year. For example, the sample of Instagram posts was extracted during the month of May, which was Skin Cancer Awareness Month. Posts in Winter months may look different than those in spring and summer months, and we suggest that future research consider such possible issues of seasonality.

### Strengths and Future Research Recommendations

Despite these limitations, this study has several strengths and implications. The study findings illuminated a paucity of medically credible sources related to skin cancer on Instagram, at least in our sample. For example, a relatively high percentage of the sources of skin cancer content in our sample were of a nonmedical nature. In addition, almost 90% of the skin cancer information in this sample was not backed by any credible citations, which is concerning. As linking health behavior to misinformation is difficult to observe, further research is needed to confirm that the spread of misinformation among users could lead to poor decision-making as it relates to skin cancer prevention, as well as public confusion [25,28].

Although Instagram is the most viewed social media platform regarding skin cancer [7], there are only a handful of studies addressing the potential of Instagram as a venue to reach out, educate, and increase public awareness regarding skin cancer. In this study, the results exposed the dominance of nonmedical skin cancer–related content on Instagram, raising concerns about the presence of misleading information. Future studies should deeply analyze content accuracy, what type of misinformation is currently spreading, and whether it is confined to causes or treatment or prevention methods. Furthermore, the findings from this study call on the medical community and public health officials to collaborate and provide leadership on these platforms. In addition, we ask them to work on more innovative and interactive techniques to engage with users and address their specific needs regarding skin cancer information, especially on key issues such as tanning bed use and avoiding other UV radiation.

### Thoughts on Possible Interventions

As a possible intervention strategy, given that influencers were prevalent in our sample, medical experts could partner with celebrities and influencers to lead awareness campaigns on skin cancer. In considering the overall presence of physicians on social media, dermatologists are among the top [29]. In fact, there are a few examples of medical influencers who have had a positive impact on skin cancer awareness. For example, Dr Sandra Lee, also known as Dr Pimple Popper [29], has had a strong reach with millions of followers and subscribers on Instagram and YouTube.

An example of a nonmedical celebrity who has raised awareness about skin cancer is Australian actor Hugh Jackman, who was diagnosed 6 times with basal cell carcinoma on his nose, which required a surgical treatment. Jackman took advantage of his popularity and used his social media platforms to advise followers regarding the risks of exposure to the sun by openly sharing his experience and medical process. This led to increased public awareness, verified by a spike in online searches for “Basal Cell Carcinoma” at the time of his skin cancer–related post [30].

### Conclusions

In summary, this study’s findings highlight the potential role of Instagram as a platform for improving awareness of skin cancer risks and the benefits of prevention practices. As skin cancer remains one of the most common cancers in the United States [1,2], public health organizations must adopt innovative ways to educate and engage with priority populations via social media platforms. Given the popularity of social media and its potential as a cost-effective method for the dissemination of health information [31], it is crucial to study the users’ engagement patterns and conversational themes around skin cancer across additional social media platforms to better understand the landscape of skin cancer narratives and how it can be used to guide the creation of customized messages and interventions that target user needs. We believe that social media is one of the most promising venues for researchers and dermatologists to dedicate their efforts and presence that can widely reach the public to educate about skin cancer and empower skin cancer prevention.

## Appendix

Multimedia Appendix 1 Codebook.

## Abbreviations

RQ: research question
UV: ultraviolet
