# Supplementary material for: Skin Cancer Narratives on Instagram: Content Analysis
Source: JMIR Infodemiology. 2022 Jun 2;2(1):e34940. doi: 10.2196/34940 (PMC9987185; doi:10.2196/34940)
Supplement: Multimedia Appendix 1 [file infodemiology_v2i1e34940_app1.docx]

**Appendix: Codebook**

| Element | Variable name | Coded theme | Code | Definition | Source |  |
| --- | --- | --- | --- | --- | --- | --- |
| Inclusion | Include | Inclusion | No = 0  Yes = 1 | Does the post meet inclusion criteria? (English language, U.S. based, and mentions skin cancer in humans in some capacity anywhere in the post or image (this can be in an Infographic and not specified in text). |  |  |
| Exclusion criteria | ExcludeReason | Exclusion | Non-U.S based = 1  Skin cancer in animals = 2  Other cancer related = 4  Other = 5 | *Display if Include = 0* |  |  |
| RQ1: What are the source and content characteristics of posts related to skin cancer? | | | | | Adapted from Tang & Park (2017) |  |
| Source characteristics  (Profile Type) | ProfileType | Profile Type | Individual = 1  Organization = 2  Other (Please describe) = 3 | 1. Profile represents a single person, not an organization or a cause. 2. Profile represents an organization, not a single person. 3. Profile represents a profile other than an individual or organization | Walsh-Buhi et al (2021) |  |
| Source location | WhatLoc  GeotaggedTXT | Location | No=0  Yes=1  If yes, fill in GeotaggedTXT with exact geotag | Location is specified anywhere in the post, either geotagged or referenced in text. | New |  |
| Source characteristics – Organization  (When referencing profile bio, you may click link to confirm organization classification)  *Check all that apply* | Business | Business Entity | No = 0  Yes = 1 | *Display if ProfileType = 2*  Profile explicitly mentions a company, franchise, business, store, product, or service. ‘Treating patients’ qualifies as a service. The word ‘.com’ may be a qualifier. Exclude non-profit and government. | Walsh-Buhi et al (2021) |  |
|  | Nonprofit | Advocacy/Non-Profit Group | No = 0  Yes = 1 | *Display if ProfileType = 2*  Profile represents an organization that advocates for a specific cause (‘skin cancer prevention’). The word ‘foundation’, mention of 501c3, ‘non-profit’, and ‘.org’ are qualifiers. Exclude business and government. | Walsh-Buhi et al (2021) |  |
|  | Government | Government | No = 0  Yes = 1 | *Display if ProfileType = 2*  Profile represents city, state, or federal government agency. “.gov” is a qualifier. Exclude business and government. | Walsh-Buhi et al (2021) |  |
|  | News | News/Media Organization | No = 0  Yes = 1 | *Display if ProfileType = 2*  Profile represents a news organization or type of media outlet. Media can be television, print, or digital. Mention of ‘Network’ or ‘providing news’ isn’t enough to qualify on its own. (e.g., CNN, New York Times, Wall Street Journal) | Walsh-Buhi et al (2021) |  |
|  | School | School | No = 0  Yes = 1 | *Display if ProfileType = 2*  Profile represents a school or school district. Cannot be an online or for-profit school. The word ‘affiliate’ of X school and ‘.edu’ are qualifiers. | Walsh-Buhi et al (2021) |  |
|  | HealthInfoAdvoc | Health Information Provider | No = 0  Yes = 1 | *Display if ProfileType = 2*  Profile cites the organization’s purpose as a form of: health education, promotion, advocacy, communication, or new updates. | Walsh-Buhi et al (2021) |  |
|  | CareOrg | Healthcare Organization | No = 0  Yes = 1 | *Display if ProfileType = 2*  Profile represents a healthcare establishment that is tangible. (Hospital, clinic, dermatological practice). This can also be coded as Business or Non-Profit in addition to CareOrg. | Walsh-Buhi et al (2021) |  |
|  | NonHealthAdvoc | Non-Health Advocacy Group | No = 0  Yes = 1 | *Display if ProfileType = 2*  Profile represents an advocacy group that is non-health related (law, etc.). | Walsh-Buhi et al (2021) |  |
| Source characteristics – Individual  *Check all that apply* | Parent | Mother/Father | No = 0  Yes = 1 | *Display if ProfileType = 1*  Any mention on user profile of being a mother or father. | Walsh-Buhi et al (2021) |  |
|  | Child | Son/Daughter` | No = 0  Yes = 1 | *Display if ProfileType = 1*  Any mention on user profile of being a son or daughter. | Walsh-Buhi et al (2021) |  |
|  | Journalist | Journalist | No = 0  Yes = 1 | *Display if ProfileType = 1*  Any mention on user profile of a news organization, or to being a press member/journalist. | Walsh-Buhi et al (2021) |  |
|  | Physician | Doctor | No = 0  Yes = 1 | *Display if ProfileType = 1*  Any mention on user profile of being a medical doctor, M.D, D.O, or medical school resident. | Walsh-Buhi et al (2021) |  |
|  | Derm | Dermatologist | No = 0  Yes =1 | *Display if ProfileType = 1*  Any mention on user profile of being a dermatologist. | New |  |
|  | Esthetician | Esthetician | No = 0  Yes =1 | *Display if ProfileType = 1*  Any mention on user profile of being an esthetician. | New |  |
|  | Epi | Epidemiologist | No = 0  Yes = 1 | *Display if ProfileType = 1*  Any mention on user profile of being an Epidemiologist (not doctor, health educator or health worker. | Walsh-Buhi et al (2021) |  |
|  | HlthEduc | Health educator | No = 0  Yes = 1 | *Display if ProfileType = 1*  Any mention on user profile of being a health educator (not doctor or nurse). | Walsh-Buhi et al (2021) |  |
|  | HlthWorker | Nurse/Other Health Worker | No = 0  Yes = 1 | *Display if ProfileType = 1*  Any mention on user profile of being a nurse or health worker of any kind (not doctor or health educator). | Walsh-Buhi et al (2021) |  |
|  | PublicFigure | Celebrity/influencer | No = 0  Yes = 1 | *Display if ProfileType = 1*  User profile is categorized as a public figure. Minimum of 20,000 followers. Any verified account would be included. | New |  |
|  | BizOwner | Business Owner/ representative | No = 0  Yes = 1 | *Display if ProfileType = 1*  Any mention on user profile of being a business owner, but the account appears to be created in their personal capacity. | New |  |
| Content - Information about skin cancer | Photo | Photo provided as media | No = 0  Yes = 1 | Post includes some sort of photo (non-moving photo image, snapshot). | Walsh-Buhi et al (2021) |  |
|  | Infographic | Infographic provided as media | No = 0  Yes = 1 | Post includes some sort of infographic (photos, graphics, or illustrations with factual information included on them, (e.g., charts). | Walsh-Buhi et al (2021) |  |
|  | Video | Video provided as media | No = 0  Yes = 1 | Post includes some sort of video (e.g., Boomerangs, gifs, IGTV). | Walsh-Buhi et al (2021) |  |
|  | Likes |  | Mean  Median  Range |  | Basch & Hillyer (2020) |  |
|  | Comments |  | Mean  Median  Range |  | Adapted from Basch & Hillyer (2020) |  |
|  | RaceTag; RaceTagTXT |  | No = 0  Yes = 1  If yes, copy and paste | Post includes race-associated tags (e.g., #Black, #Latino). | Walsh-Buhi et al (2021) |  |
|  | GenderTag  MaleTagTXT  FemaleTagTXT  OtherGenderTXT |  | No = 0  Yes= 1 | Post includes gender-associated tags (e.g., "man," "boy," "woman," "girl," "trans," "transgirl,"). If yes, copy and paste text.. | Adapted from Muralidhara & Park (2018) |  |
| Content- Photo characteristics | BP_None  BP_Scalp  BP_Face  BP_Nose  BP_Ears  BP_Lips  BP_Neck  BP_ChestBP_Arms  BP_Hands  BP_Legs | Body Part | No = 0  Yes = 1 | Image displays skin cancer on a specific body part, i.e. scalp, face, nose, tops of the ears, lips, neck, chest, arms, backs of the hands, legs. | New |  |
|  | | Model | Model | No = 0  Yes = 1 | Image displays a model or models in photo. | New |
|  | Age_Child  Age_Adolescent  Age_EarlyAdult  Age_Adult  Age_OlderAdult  Age_DontKnow | Age of model | Unable to tell = 0  Child = 1  Adolescent = 2  Early Adult = 3  Adult = 4  Older Adult = 5  Multiple ages = 6 | *Display if Model=1*  If multiple people are depicted, check all that apply. If only one person appears, please estimate or put "unable to tell," if applicable. Only code if you can see the full face and it is unobstructed, otherwise select "unable to tell.")  Person depicted appears to be:  Child = 0-12  Adolescent = 13-17  Early adult = 18 – 29  Adult = 30-64  Older Adult =65 and older  If multiple people in the picture with varying age groups, select “Multiple ages” | Adapted from Park et al (2018) |  |
| RQ2: To what extent are different types of skin cancer covered on Instagram? | | | | | Adapted from Tang & Park (2017) |  |
|  | SC_Type_None  SC_Type_Mel  SC_Type_Basal  SC_Type_Squam  SC_Type_Other | Skin Cancer Type | No Type Specified = 0  Melanoma = 11  Basal Cell Carcinoma = 2  Squamous Cell Carcinoma = 3  Other = 4 | The post contains skin cancer type specific associated tag ( ie, “#melanoma,” “basalcellcarcinoma”). Or any mention within post text or on the image. | Adapted from Jhawar & Lipoff JB. |  |
| RQ3: Cause and solution framing of skin cancer in terms of cause, treatment and prevention? | | | | | Adapted from Tang & Park (2017) |  |
| Content- Risk factors (cause) | SC_Risk | Skin Cancer Risk | No = 0  Yes = 1 | Does post discuss skin cancer risk factors? (There should be an explicit mention of risk due to sun exposure or other risks--tanning or genetic predisposition.) |  |  |
|  | Risk_Tanning | Artificial Tanning | No = 0  Yes = 1 | *Display if SC_Risk=1*  Post mentions artificial tanning as a risk factor to skin cancer | Basch & Hillyer (2020) |  |
|  | Risk_Sun | Sun Exposure | No = 0  Yes = 1 | *Display if SC_Risk=1*  Post mentions sun exposure as a risk factor to skin cancer | Basch & Hillyer (2020) |  |
|  | Risk_Genetics | Genetics | No = 0  Yes= 1 | *Display if SC_Risk=1*  Post mentions genetics as a risk factor to skin cancer. | Adapted from Tang & Park (2017) |  |
| Content- Prevention  Primary | Prevention | Prevention | No = 0  Yes = 1 | Does post discuss skin cancer prevention methods? |  |  |
|  | SelfTan | Promotes self-tanner | No = 0  Yes = 1 | *Display if Prevention=1*  Post promotes use of self-tanning products as a form of skin cancer prevention. | Basch & Hillyer (2020) |  |
|  | Sunscreen | Promotes sunscreen | No = 0  Yes = 1 | *Display if Prevention=1*  Post promotes wearing sunscreen as a form of skin cancer prevention. | Basch & Hillyer (2020) |  |
|  | ProtectGear | Promotes protective clothing/gear | No = 0  Yes = 1 | *Display if Prevention=1*  Post promotes wearing protective clothing as a form of skin cancer prevention. | Tang & Park (2017) |  |
|  | TanBeds | Promotes avoiding tanning beds | No = 0  Yes = 1 | *Display if Prevention=1*  Post promotes not using tanning beds as a form of skin cancer prevention. | Tang & Park (2017) |  |
|  | Shade | Promotes seeking shade | No = 0  Yes = 1 | *Display if Prevention=1*  Post promotes staying out of the sun as a form of skin cancer prevention. | Tang & Park (2017) |  |
| Content- Prevention  Secondary | WarningSign | Mentions warning signs (ABCDEs) | No = 0  Yes = 1 | Post mentions warning signs of skin cancer (Asymmetrical, Borders are Irregular, Color is not even, Diameter is large, Evolving) | Basch & Hillyer (2020) |  |
|  | SelfExam | Mentions self-examination | No = 0  Yes = 1 | Post promotes self-examination to detect/prevent skin cancer. | Tang & Park (2017) |  |
|  | DocExam | Mentions examination by a dermatologist/ doctor | No = 0  Yes = 1 | Post promotes getting check-ups from a dermatologist or a doctor to detect/prevent skin cancer. | Tang & Park (2017) |  |
| RQ4: What is the extent to which Instagram posts on skin cancer addressed the susceptibility and severity of skin cancer, benefits and barriers associated with diagnosis, prevention and treatment, cue to action, and readers’ self- efficacy? | | | | | Adapted from Tang & Park (2017) |  |
| Content – Diagnosis, treatment, and prevention | Diagnosis | Diagnosis | No = 0  Yes = 1 | Discusses skin cancer diagnostic methods (ie. Skin biopsy, punch biopsy) | Basch & Hillyer (2020) |  |
|  | Treatment | Treatment | No = 0  Yes = 1 | Mentions skin cancer treatment. (ie. Radiation therapy, chemotherapy, herbal remedies) | Basch & Hillyer (2020) |  |
|  | AltTreat | Promotes alternative treatment | No = 0  Yes = 1 | Mentions alternative skin cancer treatment (ie. herbal remedies, homemade remedies, foods) either in text or reflected in the image. | Basch & Hillyer (2020) |  |
|  | TradTreat | Promotes traditional biomedical treatment | No = 0  Yes = 1 | Mentions traditional biomedical skin cancer treatment (ie. Surgery (Mohs surgery), biopsy, radiation therapy, chemotherapy) either in the text or reflected in the image. | Tang & Park (2017) |  |
|  | Prevention | Prevention | No = 0  Yes = 1 | Post mentions skin cancer prevention methods (ie. wearing sunscreen, staying out of the sun) | Basch & Hillyer (2020) |  |
| Content- Prevalence | Prevalence | Discusses the prevalence/ susceptibility of skin cancer | No = 0  Yes = 1 | Post discusses the prevalence of skin cancer, ie. “skin cancer is the most common type of cancer in the United States,” “young women under 29 years are at increased risk for skin cancer”) | Tang & Park (2017) |  |
| Content- Severity | Seriousness | Discusses how serious skin cancer could be | No = 0  Yes = 1 | Post discusses the seriousness of skin cancer in terms of medical consequences (death), financial consequences (high cost of treatment), or other types of consequences. | Tang & Park (2017) |  |
| Content- Benefits | PrevBenefit | Discusses the benefit of a prevention method | No = 0  Yes = 1 | *Display if Prevention=1*  The post discusses the benefits of a prevention method (e.g., wearing sunscreen decreases signs of aging, hydrates, improves wrinkles, etc.). | Adapted from Tang & Park (2017) |  |
|  | DiagBenefit | Discusses the benefit of a diagnostic method | No = 0  Yes = 1 | *Display if Diagnosis=1*  The post discusses the benefits of diagnostic methods (ie. the punch biopsy method yields deeper tissue). | Adapted from Tang & Park (2017) |  |
|  | TreatBenefit | Discusses the benefit of a treatment | No = 0  Yes = 1 | *Display if Treatment=1*  The post discusses the benefits of a treatment (ie. surgery is the most effective skin cancer treatment). | Adapted from Tang & Park (2017) |  |
| Content - Barriers | DiagBarrier | Discusses the barriers of diagnostic methods | No = 0  Yes = 1 | *Display if Diagnosis=1*  The post discusses barriers to diagnostic skin cancer methods (ie. lack of knowledge on what skin cancer looks like, lack of skilled or trained physicians). | Adapted from Tang & Park (2017) |  |
|  | TreatBarrier | Discusses barriers of treatment | No = 0  Yes = 1 | *Display if Treatment=1*  The post discusses barriers to treatment of skin cancer (ie. treatment is too expensive). | Adapted from Tang & Park (2017) |  |
|  | PrevBarrier | Discusses barriers of prevention | No = 0  Yes = 1 | *Display if Prevention=1*  The post discusses barriers to the prevention of skin cancer (ie. lack of knowledge on prevention methods). | Adapted from Tang & Park (2017) |  |
| Content- HBM | AdoptBehavior | Includes a cue to action | No = 0  Yes = 1 | The post includes information that urges readers to adopt a certain behavior (eg. “protect yourself”, “always wear sunscreen”). | Adapted from Tang & Park (2017) |  |
| Content - Source attribution | Cite | Citation included | No = 0  Yes = 1 | the post cites a source for skin cancer information? (e.g., CDC, WHO, a doctor, peer-review journal, etc. It is okay to indicate "yes," if the post originator is citing itself, as long as it is a clear citation.) | Walsh-Buhi et al (2021) |  |
|  | GovInfo | Cites the CDC | No = 0  Yes = 1 | *Display if Cite=1*  The post cites information from the CDC or other federal level or foreign equivalent sources. | Walsh-Buhi et al (2021) |  |
|  | DoctorInfo | Cites a doctor | No = 0  Yes = 1 | *Display if Cite=1*  The post cites information from a medical doctor (e.g., Dr. Martinez, a local pediatrician). Announcing a talk by a doctor is not sufficient. | Walsh-Buhi et al (2021) |  |
|  | PoliticalInfo | Cites political officials or political organizations | No = 0  Yes = 1 | *Display if Cite=1*  The post cites information from government officials (e.g., senators, governors, representatives) or from political organizations (e.g., Focus on the Family). | Walsh-Buhi et al (2021) |  |
|  | CancerOrgInfo | Cites cancer organizations | No = 0  Yes = 1 | *Display if Cite=1*  The post cites information from cancer organizations (e.g., American Cancer Society). | Walsh-Buhi et al (2021) |  |
|  | ResearchInfo | Cites research community | No = 0  Yes = 1 | *Display if Cite=1*  The post cites information from a member of the research community (e.g., researchers, scientists, epidemiologists, or professors) . | Walsh-Buhi et al (2021) |  |
|  | OtherWebInfo | Cites WebMD or other health/web source | No = 0  Yes = 1 | *Display if Cite=1*  Other post source such as WebMD, Mayo Clinic, etc. (not CDC or above). | Walsh-Buhi et al (2021) |  |
|  | CelebrityInfo | Cites celebrity | No = 0  Yes = 1 | *Display if Cite=1*  The post cites information from a celebrity. | Walsh-Buhi et al (2021) |  |
|  | HealthDeptInfo | Cites state or local health department | No = 0  Yes = 1 | *Display if Cite=1*  The post cites information from a state or local health department. | Walsh-Buhi et al (2021) |  |
|  | WHOInfo | Cites WHO | No = 0  Yes = 1 | *Display if Cite=1*  The post cites information from the World Health Organization (WHO) | Walsh-Buhi et al (2021) |  |
| Content - Personal account | Personal | Personal account | No = 0  Yes = 1 | Post has presence of personal account from individuals who discussed a firsthand experience with skin cancer (Only from individual posters) | Walsh-Buhi et al (2021) |  |
|  | Hashtag |  |  | Copy and paste all hashtags from the post. (Check also for account owner comments that include additional hashtags; do not include comments by others.) | Walsh-Buhi et al (2021) |  |
| RQ5: What are characteristics of the contents of the top 10% most liked posts on skin cancer within the sample? | | | | | Adapted from Tang & Park (2017) |  |
